# Healable Dielectric Elastomer Actuator With Premature Breakdown Warning Function for Information Transmission Encryption and Human‐Machine Interaction

**DOI:** 10.1002/advs.202505829

**Published:** 2025-06-25

**Authors:** Yanze Liu, Shilin Luo, Jiawei Zhao, Lei Xu, Zhong‐Zhen Yu, Dan Yang

**Affiliations:** ^1^ State Key Laboratory of Organic‐Inorganic Composites College of Materials Science and Engineering Beijing University of Chemial Technology Beijing 100029 China; ^2^ Center for Nanomaterials and Nanocomposites College of Materials Science and Engineering Beijing University of Chemical Technology Beijing 100029 China

**Keywords:** actuator, compliant electrode, dielectric elastomer, healable, ZnS:Cu

## Abstract

Dielectric elastomer actuators (DEAs), regarded as artificial muscles, are used as actuators or sensors in artificial visual systems, haptic equipment, and human‐robot interaction. However, their inherent vulnerability related to mechanical damage and electrical breakdown seriously limits their safe and long‐term service. Herein, a healable dielectric elastomer (PHT‐ZnS:Cu) is acquired for premature breakdown warning by synthesising a healable poly(dimethylsiloxane) based on reversible imine bonding and hydrogen bonding and subsequent incorporation of electroluminescent ZnS:Cu particles. Moreover, LiTFSI ionic liquids are introduced into the healable poly(dimethylsiloxane) to prepare a transparent healable compliant electrode (PHT‐IL) that does not obscure the light emission of PHT‐ZnS:Cu dielectric elastomer. Then, an integral healable DEA (ISDEA) with an excellent interfacial interaction is constructed by coating two sides of PHT‐ZnS:Cu with PHT‐IL. The optimized ISDEA displays an actuated strain of 17.0% at 15 kV mm^−1^ with a healing efficiency of 91.9%. The integrated ISDEAs are used as soft anti‐counterfeiting labels and soft intelligent keyboards for information transmission, encryption, and human‐machine interaction, showing prospective applications in the realm of data leakage prevention and personal privacy protection.

## Graphical abstract

An integral healable dielectric elastomer actuator (ISDEA) with a premature breakdown warning function is prepared based on the combination of electroluminescent dielectric elastomer and transparent compliant electrodes. Several ISDEAs are combined to construct soft anti‐counterfeiting labels and soft intelligent keyboards, expanding their potential application in data leakage prevention and personal privacy protection.

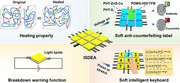

## 1 Introduction

Dielectric elastomer actuators (DEAs), consisting of a thin dielectric elastomer film sandwiched between two compliant electrodes, produce deformation in response to electrical stimulation.^[^ ^1^, ^2^, ^3^ ^]^ The good characteristics of DEAs in terms of large actuation, rapid response, and good conformability^[^ ^4^, ^5^, ^6^ ^]^ make them prospective for use as artificial muscles with function of actuators or sensors in visual systems,^[^ ^7^, ^8^, ^9^ ^]^ braille displays,^[^ ^10^, ^11^ ^]^ and human‐robot interaction.^[^ ^12^, ^13^ ^]^ Unfortunately, the compliant electrodes and dielectric elastomers are prone to mechanical damage during repetitive use or under high mechanical stress.^[^ ^14^, ^15^ ^]^ In addition, the required high electric field (≈100 kV mm^−1^) for driving DEAs usually results in their electrical breakdown.^[^ ^16^ ^]^ Moreover, the mechanical and electrical damages of DEAs significantly decrease their service lifetime and produce substantial waste that contradicts sustainable development.

These issues can be solved by preparing DEAs with healable properties, which can be accomplished by introducing reversible non‐covalent bonds (e.g., hydrogen bonds,^[^ ^17^, ^18^, ^19^ ^]^ metal‐coordination,^[^ ^20^, ^21^ ^]^ and electrostatic interaction^[^ ^22^, ^23^ ^]^) or covalent bonds (e.g., Diels–Alder reactions,^[^ ^24^, ^25^ ^]^ disulfide bonds,^[^ ^26^, ^27^ ^]^ and boronic ester bonds^[^ ^28^, ^29^ ^]^) into polymer chains. The reconstructed bonds would repair defects or voids in the damaged elastomers, making DEAs reusable. Nevertheless, most healable DEAs focus on the preparation of healable dielectric elastomers or healable compliant electrodes independently.^[^ ^30^, ^31^, ^32^ ^]^ Since damage to DEAs during operation is usually holistic, it is therefore imperative to design integral healable DEAs (ISDEAs) with both the dielectric film and compliant electrodes characterized by healing abilities.

In a previous study, ISDEA was prepared by introducing conductive polyaniline blocks into the poly(dimethylsiloxane) (PDMS) backbones.^[^ ^33^ ^]^ The presence of hydrogen bonds endows the as‐prepared ISDEA with an actuated strain of 1.62% after healing from mechanical damage. However, the healing efficiency based on the maximum actuated strain is only 22.6%. Moreover, the actuation performance of the as‐prepared ISDEA after electrical damage has not been studied. In previous researches, although some breakdown warning systems have been developed to improve the safety of devices prone to electrical breakdown,^[^ ^34^, ^35^ ^]^ they are not yet implemented in DEAs. Based on electroluminescence (EL), ZnS:Cu particles emit light in response to an electric field or current.^[^ ^36^, ^37^, ^38^ ^]^ As a result, dispersing ZnS:Cu particles in dielectric elastomer matrices could provide the function of premature breakdown warning for DEAs. Under these circumstances, the application of a voltage close to the breakdown strength of DEAs would accumulate electric charge around the defects, significantly enhancing the local electric field and inciting the ZnS:Cu particles around the defects in dielectric elastomers to emit light. Accordingly, the operator can turn off the voltage timely when seeing the light emitted by DEAs to avoid the occurrence of electrical breakdown.

Nevertheless, compliant electrodes are often fabricated by dispersing conductive fillers (e.g., MXene,^[^ ^39^, ^40^ ^]^ carbon nanotubes,^[^ ^41^, ^42^ ^]^ and graphene,^[^ ^43^, ^44^ ^]^) within silicone oil, inevitably challenging the observation of the light‐emitting phenomenon by ZnS:Cu particles in dielectric elastomers. Therefore, it is expected that prepare transparent compliant electrodes with excellent healing properties to ensure the function of premature breakdown warning of DEAs. Ionic liquids are good candidates for conductive fillers to prepare flexible transparent electrodes owing to their excellent flexibility, good electrical conductivity, and transparency.^[^ ^45^, ^46^, ^47^, ^48^, ^49^ ^]^

**Figure 1:**
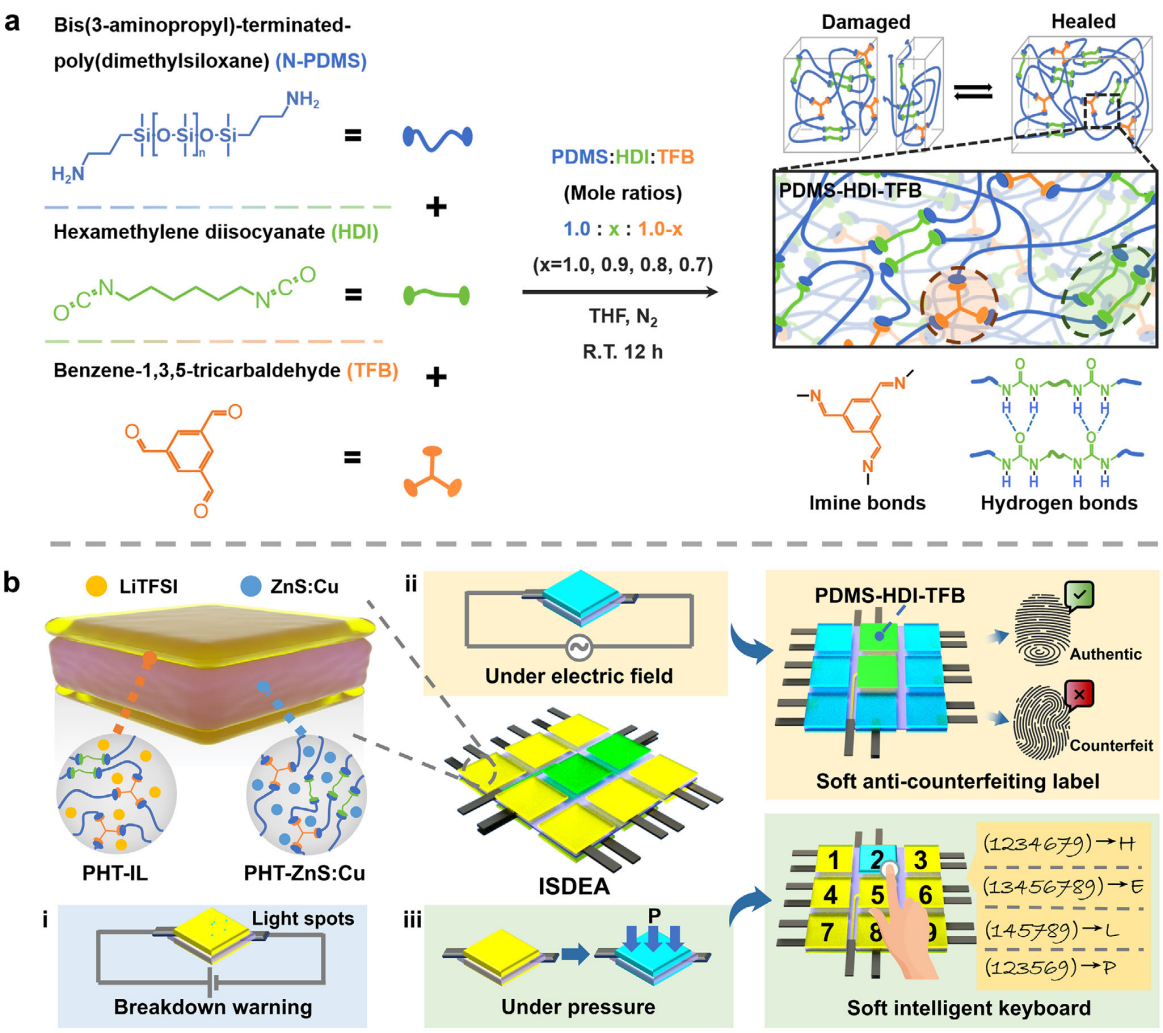
a) The illustration of the synthesis process of PDMS‐HDI‐TFB. b) Schematic diagram of i) ISDEA with premature breakdown warning function and its application as ii) a soft anti‐counterfeiting label and iii) a soft intelligent keyboard.

Herein, a healable poly(dimethylsiloxane) elastomer (PDMS‐HDI‐TFB) with reversible imine bonds and multiple hydrogen bonds was first synthesised by a simple one‐pot polycondensation reaction among the amino‐modified poly(dimethylsiloxane) (N‐PDMS), 1,6‐diisocyanatohexane (HDI), and 1,3,5‐benzenetricarboxaldehyde (TFB), denoted as PDMS‐HDI‐TFB (**Figure** **1**a; Figure S1, Supporting Information). Subsequently, ZnS:Cu particles were incorporated into the PDMS‐HDI‐TFB to prepare a dielectric elastomer composite with premature breakdown warning function (PHT‐ZnS:Cu). Meanwhile, lithium bis(trifluoromethanesulfonyl)imide (LiTFSI) ionic liquids were dispersed in the PDMS‐HDI‐TFB to form a transparent compliant electrode (PHT‐IL), followed by coating on both sides of PHT‐ZnS:Cu to construct an ISDEA (Figure 1b.i). The application of an electric field close to the breakdown strength induces light emission in the ISDEA as an electrical breakdown warning signal. Under an electric field of 15 kV mm^−1^, an actuated strain of 17.0% is obtained, with a healing efficiency of 91.9%. By encoding the arrangement of PHT‐IL and PDMS‐HDI‐TFB, advanced ISDEAs arrays can be constructed as a soft anti‐counterfeiting label with a function to prevent infringement (Figure 1b.ii). Based on the capacitance changes, the ISDEAs arrays may also be used as a soft intelligent keyboard (Figure 1b.iii) with promising application in human‐computer interaction for information transmission and emotion expression.

## 2 Results and Discussion

**Figure 2:**
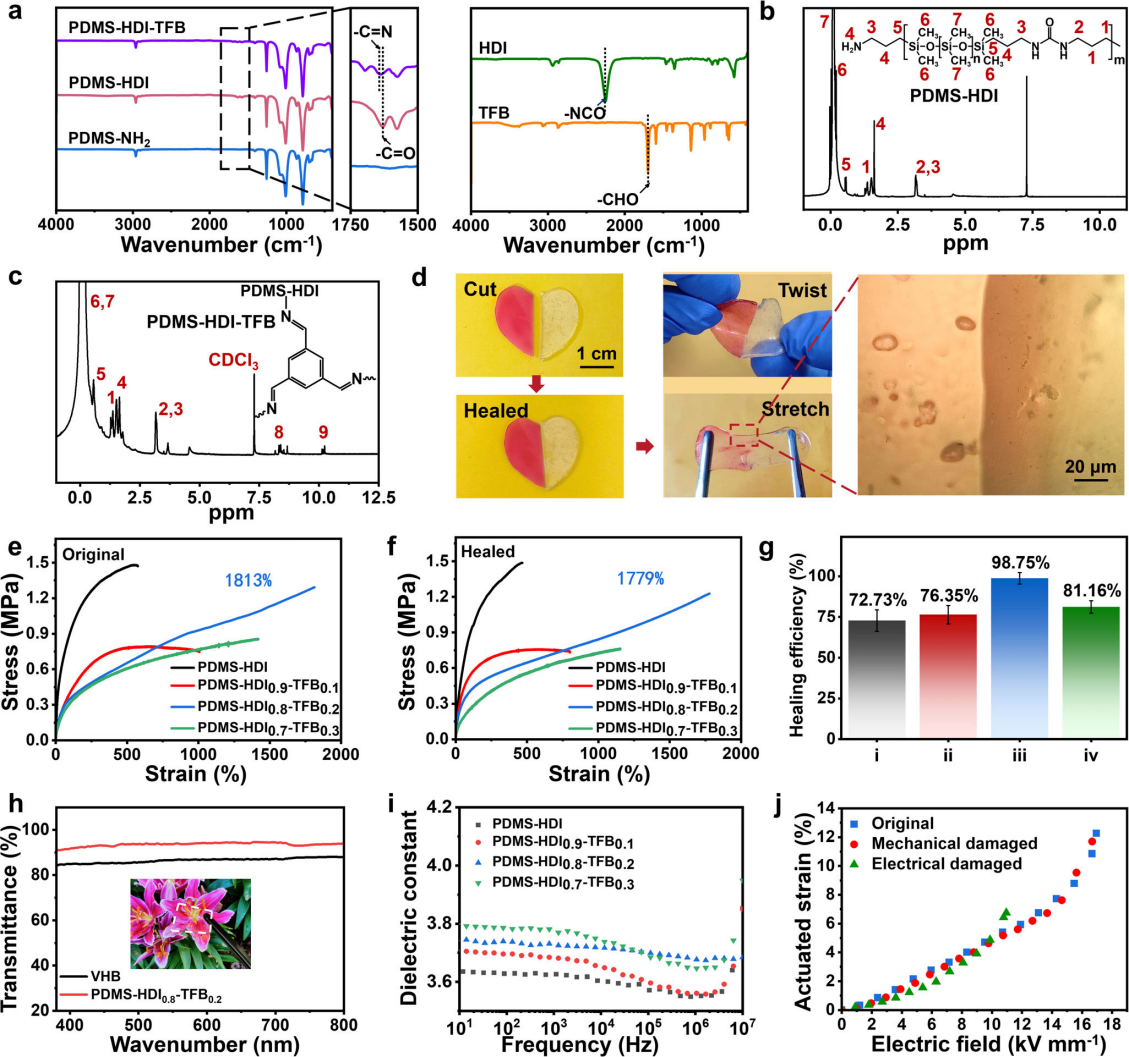
a) FT‐IR spectra of HDI, TFB, N‐PDMS, PDMS‐HDI, and PDMS‐HDI‐TFB. ^1^H NMR spectra of b) PDMS‐HDI and c) PDMS‐HDI‐TFB. d) Optical microscopy photos of a heart‐shaped PDMS‐HDI_0.8_‐TFB_0.2_ being twisted and stretched after healing. The left separated piece was dyed with oil red O. Tensile stress–strain curves of PDMS‐HDI_x_‐TFB_1‐x_ e) before mechanical damage and f) after healing. g) Healing efficiencies based on elongation at break of i) PDMS‐ HDI, ii) PDMS‐HDI_0.9_‐TFB_0.1_, iii) PDMS‐HDI_0.8_‐TFB_0.2_, and iv) PDMS‐HDI_0.7_‐TFB_0.3_. h) UV–vis transmission spectra of PDMS‐HDI_0.8_‐TFB_0.2_ and VHB. The inset photograph shows the transparent feature of PDMS‐HDI_0.8_‐TFB_0.2_. i) Dielectric constant of PDMS‐HDI_x_‐TFB_1‐x_ versus frequency. j) The actuated strains of the original and healed DEA, consisting of PDMS‐HDI_0.8_‐TFB_0.2_ and carbon grease after mechanical damage and electrical damage.

In this work, an ISDEA with a premature breakdown warning function was prepared. The successful synthesis of the PDMS‐HDI‐TFB was confirmed by Fourier transform infrared (FT‐IR) spectroscopy. As shown in **Figure** **2**a, the asymmetrical stretching vibration peak of ‐NCO groups in HDI at 2257 cm^−1^ completely disappears, while the vibration absorption peak of C═O of urea groups appears at 1630 cm^−1^ in the PDMS‐HDI, indicating the successful reaction between ‐NH_2_ in N‐PDMS and ‐NCO in HDI. After the reaction of PDMS‐HDI with TFB, the ‐CHO peak at 1700 cm^−1^ in TFB vanishes while that of urea groups redshifts to 1646 cm^−1^ in PDMS‐HDI‐TFB, attributed to the generation of imine bonds (─C═N) between amino (‐NH_2_) groups in PDMS‐HDI and the aldehyde (‐CHO) groups in TFB.^[^ ^50^ ^]^ The detailed molecular structure of PDMS‐HDI‐TFB was identified by the ^1^H nuclear magnetic resonance (^1^H NMR) spectroscopy. In Figure 2b,c, the characteristic peak at 10.23 ppm can be attributed to the imine bonds resulting from the reaction between PDMS‐HDI and TFB. The multiple hydrogen bonds between the urea groups and the dynamic imine bonds endow the PDMS‐HDI‐TFB with excellent healing properties. After close contact at 60 °C for 3 h (Figure 2d), the pristine and dyed PDMS‐HDI_0.8_‐TFB_0.2_ (‘0.8’ and ‘0.2’ representing the mole fractions of HDI and TFB in PDMS‐HDI‐TFB, respectively) show good healing with heart‐shape formation able of twisting or stretching without breaking. Moreover, the optical microscopy images in Figure 2d reveal almost disappeared cracks in the PDMS‐HDI_0.8_‐TFB_0.2_ accompanied by color fusion, which can be ascribed to the excellent mobility of polymer chains contributing to the healing process.^[^ ^51^ ^]^

The stress–strain curves of original and healed PDMS‐HDI and PDMS‐HDI_x_‐TFB_1‐x_ are compared in Figure 2e,f. With the rise in TFB content, the elongation at break of PDMS‐HDI_x_‐TFB_1‐x_ first increases and then decreases. The largest elongation at break, reaching 1813%, is obtained by the original PDMS‐HDI_0.8_‐TFB_0.2_. The stretching and twisting processes of it are shown in Video S1. The super‐stretchability of PDMS‐HDI_0.8_‐TFB_0.2_ is linked to adequate crosslinking between polymer chains containing abundant hydrogen bonds able to maintain the network structure, as well as the presence of imine bonds dissipating energy. When the TFB content is low, there are more strong hydrogen bonds between MDI in PDMS‐HDI‐TFB elastomer, which improves the stiffness but makes the material less stretchable. This is consistent well with the Young's modulus of PDMS‐HDI_x_‐TFB_1‐x_ (Figure S2, Supporting Information). As the TFB content increases, dynamic imine bonds are increased, which help chains movement and release stress, thus improving elongation at break and healing efficiency (Figure 2g).^[^ ^52^ ^]^ However, the benzene ring of TFB affects the formation of hydrogen bonds.^[^ ^53^, ^54^ ^]^ Therefore, healing efficiency and elongation at break both show a decline in PDMS‐HDI_0.7_‐TFB_0.3_. Based on the original and healed elongation at break, a high healing efficiency of 98.75% is obtained by the PDMS‐HDI_0.8_‐TFB_0.2_. Such good healing performance can also be confirmed by the completely vanished cracks in PDMS‐HDI_0.8_‐TFB_0.2_ while they are still visible in other samples after healing at 60 °C for 3 h (Figure S3, Supporting Information).

The resilient ability of PDMS‐HDI_0.8_‐TFB_0.2_ was also evaluated, and Figure S4 (Supporting Information) shows the cyclic tensile curves of PDMS‐HDI_0.8_‐TFB_0.2_ with loading 50% strain and 100% strain after resting for different intervals. The cycle curve of PDMS‐HDI_0.8_‐TFB_0.2_ after rest for 1 min shows obvious hysteresis. The extension of the resting time results in cycle curves gradually approaching the original state, indicating re‐association of the broken hydrogen bonds and recovery of the mechanical properties of the material, beneficial for the cyclic use of PDMS‐HDI_0.8_‐TFB_0.2_‐based DEA. The excellent mechanical and healing properties of the PDMS‐HDI_0.8_‐TFB_0.2_ incited further studies as the elastomer matrices to prepare healable dielectric elastomers and healable compliant electrode composites.

As displayed by the UV–vis transmission spectrum (Figure 2h), the obtained PDMS‐HDI‐TFB is colorless and highly transparent, with an average transmittance over 93% in the visible range (380–800 nm), a value even higher than that of 3 ^m^ VHB tape (86%).

The dielectric constant and dielectric loss tangent of PDMS‐HDI_x_‐TFB_1‐x_ are presented in Figure 2i and Figure S5 (Supporting Information), respectively. From Figure 2i, it can be seen that the dielectric constant of PDMS‐HDI_x_‐TFB_1‐x_ increases with increasing content of TFB, which is mainly attributed to the reduction in hydrogen bonds, thus the free volume of the molecular chains increases. This enhanced molecular mobility may facilitate greater dipole orientation polarization under an electric field, thereby contributing to the elevated dielectric constant. Additionally, the presence of polar amino groups and urea groups endow PDMS‐HDI‐TFB with a high dielectric constant of 3.74 at 100 Hz, a value superior to that of Sylgard 184 silicone rubber (2.72 at 100 Hz). Moreover, the dielectric loss tangent of PDMS‐HDI_0.8_‐TFB_0.2_ remains low (0.001) at 100 Hz, which is beneficial for improving the electro‐mechanical conversion efficiency of DEA.^[^ ^55^, ^56^ ^]^

Afterward, commercial carbon grease was coated on both sides of PDMS‐HDI_x_‐TFB_1‐x_ to construct a DEA. The as‐constructed DEA was then actuated by a DC power. The maximum actuated strain achieved by the PDMS‐HDI_0.8_‐TFB_0.2_ with a value of 12.3% under 16.9 kV mm^−1^ (Figure S6, Supporting Information). After healing from mechanical damage followed by recoating carbon grease, the actuated strain of healed PDMS‐HDI_0.8_‐TFB_0.2_ still reaches 11.7% under 16.7 kV mm^−1^, with a healing efficiency of 95.1%. Due to the largest actuated strain under a low electrical field and the highest healing efficiency, we selected the PDMS‐HDI_0.8_‐TFB_0.2_ for further actuation experiments.

However, the maximal actuated strain of the PDMS‐HDI_0.8_‐TFB_0.2_ after healing from electrical damage reaches only 6.8%, with a healing efficiency of 55.3% (Figure 2j). Furthermore, the dielectric elastomer cannot fully recover the actuation of DEAs when suffering from electrical breakdown. The lower healing efficiency can be attributed to the residual carbon and carbides in PDMS‐HDI‐TFB generated by electric breakdown, hindering the mobility of polymer chains, decreasing the probability of unbound groups at the wound site to interact, and preventing the reformation of reversible bonds.^[^ ^57^ ^]^

**Figure 3:**
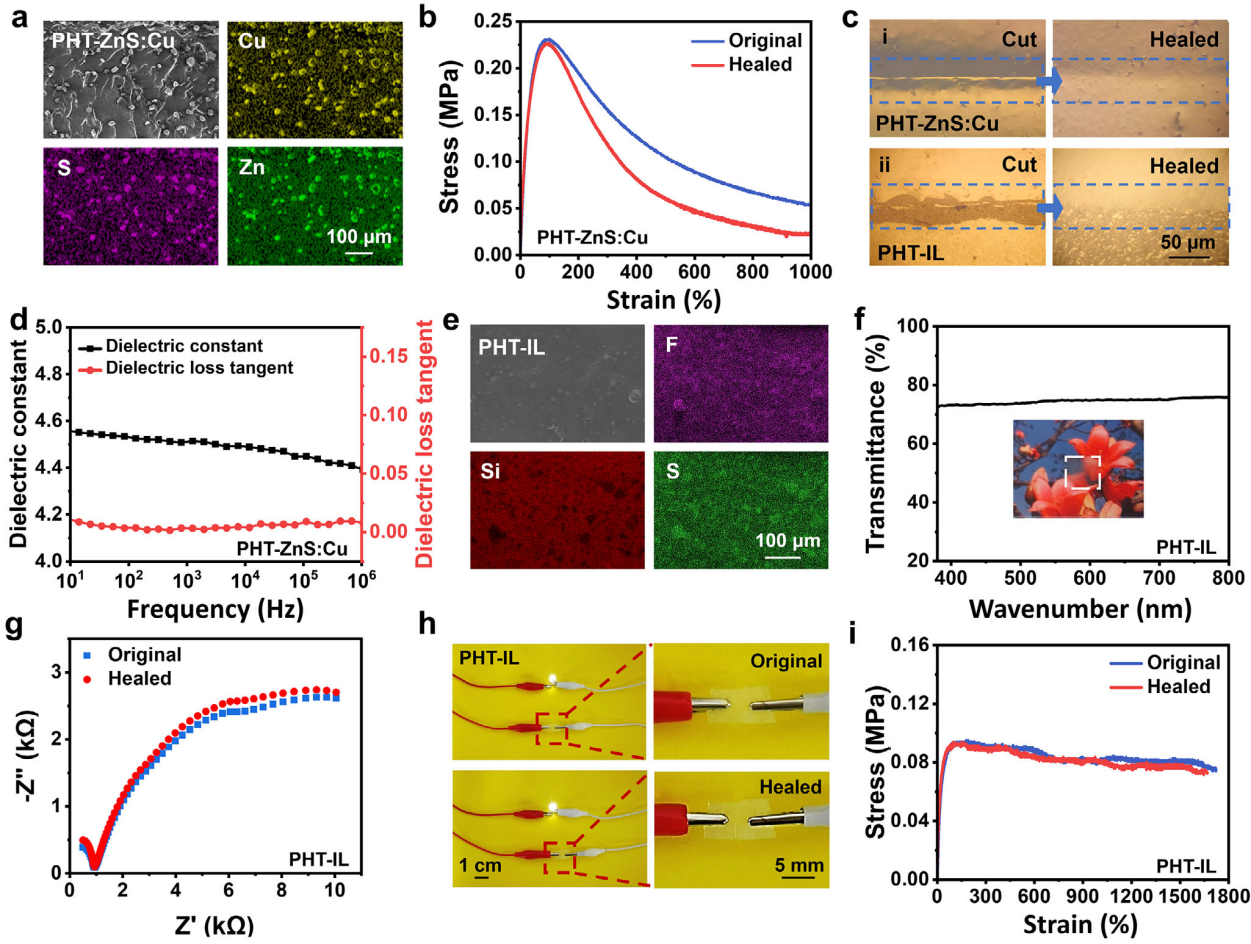
a) SEM image and EDS mappings of PHT‐ZnS:Cu. b) Tensile stress–strain curves of original and healed PHT‐ZnS:Cu. c) Optical microscopy photos of cut and healed i) PHT‐ZnS:Cu and ii) PHT‐IL. d) Dielectric constant and dielectric loss tangent of PHT‐ZnS:Cu versus frequency. e) SEM image and EDS mappings of PDSM‐IL. f) UV–vis transmission spectra of PHT‐IL. The inset photograph shows the transparency of PHT‐IL. g) Electrochemical impedance spectra of original and healed PHT‐IL. h) Digital photos of the original and healed PHT‐IL in a conductive loop with an LED bulb. i) Tensile stress–strain curves of original and healed PHT‐IL.

The most effective way to prevent DEAs from electrical breakdown is by predicting their occurrence in advance. This can be accomplished by incorporating ZnS:Cu particles in PDMS‐HDI‐TFB to prepare PHT‐ZnS:Cu dielectric elastomer with EL property. The ZnS:Cu particles exhibit an irregular morphology with an average size of 15 µm, as shown in the SEM image (Figure S7, Supporting Information). Near the occurrence of electrical breakdown, light‐emitting is generated on the PHT‐ZnS:Cu, serving as an early warning signal. As depicted by the scanning electron microscopy (SEM) image (**Figure** **3**a), the ZnS:Cu particles exhibit good dispersion in the PDMS‐HDI‐TFB matrix. The homogeneous distribution of Cu, S, and Zn elements in the EDS mappings of PHT‐ZnS:Cu also demonstrates the uniform dispersion of ZnS:Cu particles. It is worth noting that the ZnS:Cu particles decrease Young's modulus of PHT‐ZnS:Cu. According to Figure 3b, the Young's modulus of PHT‐ZnS:Cu is only ≈0.60 MPa, a value much lower than that of the PDMS‐HDI‐TFB matrix (1.23 MPa). The decreased Young's modulus of PHT‐ZnS:Cu can be assigned to the declined crosslinking density upon the addition of ZnS:Cu (from 0.67 to 0.25 mmol cm^−3^). Generally, a decrease in crosslink density will bring a decrease in Young's modulus.^[^ ^58^, ^59^ ^]^ However, the low Young's modulus benefits PHT‐ZnS:Cu in terms of high actuated strain at a low electric field, according to the Maxwell mechanism.^[^ ^55^ ^]^ In addition, the stress–strain curves of original and healed PHT‐ZnS:Cu suggest a healing efficiency of PHT‐ZnS:Cu of ≈98.0% based on the Young's modulus. Optical microscopy observations reveal completely vanished cracks in PHT‐ZnS:Cu after healing at 60 °C for 3 h (Figure 3c.i). Moreover, the dielectric constant of PHT‐ZnS:Cu increases to 4.53 at 100 Hz, a value higher than that of PDMS‐HDI‐TFB (3.74 at 100 Hz), as shown in Figure 3d. The increased dielectric constant can be attributed to the mismatch of permittivity between ZnS:Cu and PDMS‐HDI‐TFB matrix, promoting the space charge accumulation and the formation of dipoles at interfaces.^[^ ^60^, ^61^ ^]^ On the other hand, though the dielectric loss tangent of PHT‐ZnS:Cu increases to 0.003 (100 Hz), it remains at a low level.

Simultaneously, the uniform dispersion of LiTFSI in PDMS‐HDI‐TFB matrix forms a healable transparent compliant electrode, as shown in the SEM image of Figure 3e. The obtained PHT‐IL is colorless, with an average transmittance of over 74.5% in the visible range (380–800 nm) (Figure 3f). The good dispersion is further confirmed by the homogeneous distribution of F, S, and Si elements in the EDS mappings. The heteroatom oxygen (O) in PDMS‐HDI‐TFB chains with lone electron pairs, facilitates the complexation between O and lithium ions (Li⁺), resulting in a good dispersibility of LiTFSI in the PDMS‐HDI‐TFB matrix. Thus, the Li^+^ ions transport through the PDMS‐HDI‐TFB chains, culminating in efficient ionic conduction.^[^ ^47^ ^]^ Moreover, the excellent mobility of PDMS‐HDI‐TFB chains allows the reversible establishment and dissociation of Li–O coordination bonds. Consequently, the broken conductive paths can be easily reconnected, as demonstrated by the completely vanished cracks in PHT‐IL after healing at 60 °C for 30 min, as seen from the optical microscopy photos in Figure 3c.ii. Both original and healed PHT‐IL exhibit a high electric conductivity of 0.083 mS m^−1^ (Figure 3g), thus they can light the LED bulb (Figure 3h). Based on the calculation of the tensile stress–strain curves in Figure 3i, PHT‐IL recovers 99.8% of Young's modulus as compared to the original value of 1.04 MPa, indicating good healing performance.

**Figure 4:**
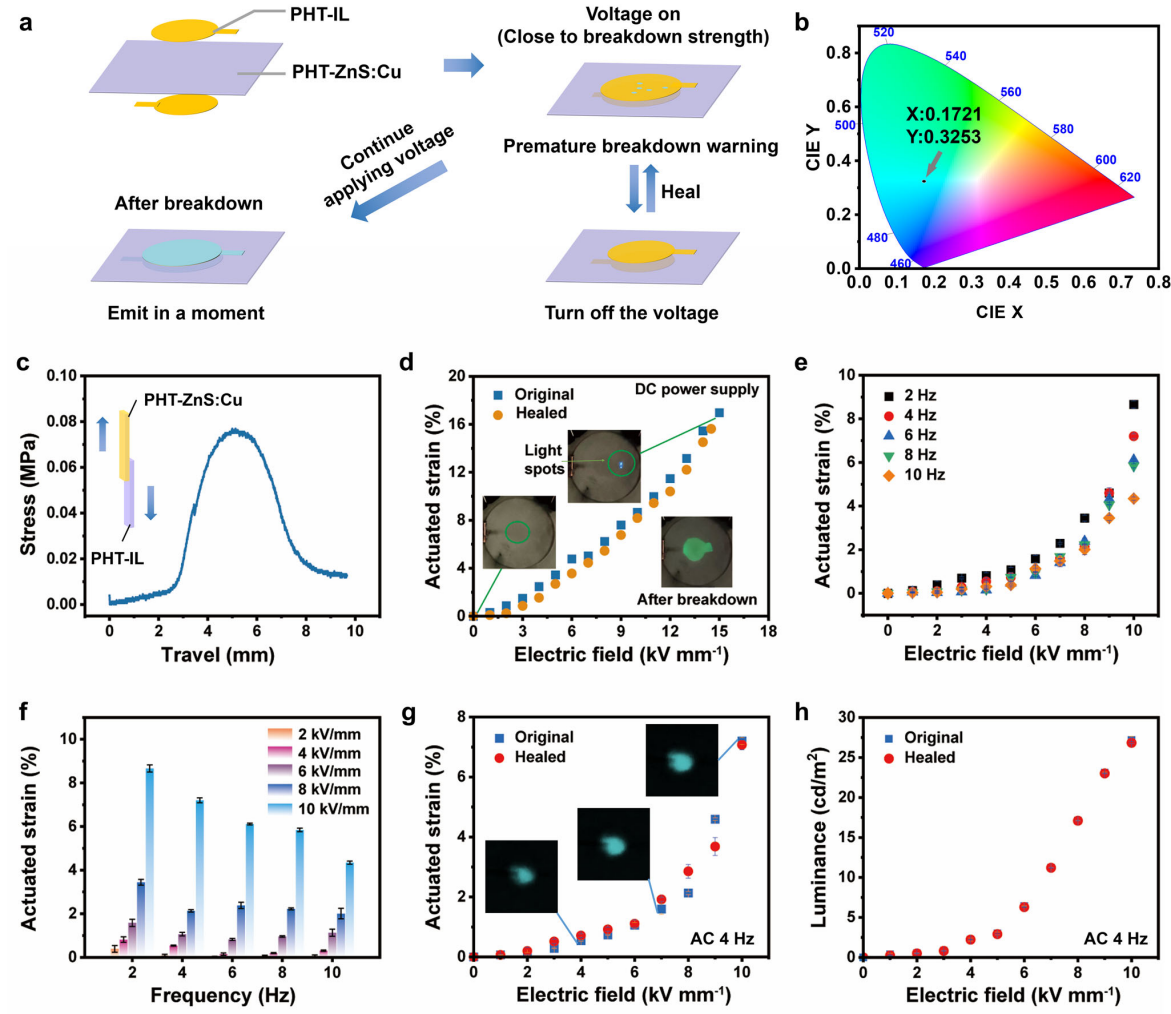
a) Illustration of construction and actuation of ISDEA. b) Color coordinates of the ISDEA on the Commission Internationale de L'Eclairage (CIE) 1931 color space. c) Adhesion test between PHT‐IL and PHT‐ZnS:Cu. d) Actuation performance of the original and healed ISDEA actuated by a DC power. The inset photographs show the actuation progress of ISDEA. e) Actuation performance of ISDEA under an AC power at different frequencies. f) Actuation performance of ISDEA under different AC electric fields. g) Actuation performance of the original and healed ISDEA under an AC power at 4 Hz. The inset photographs show the actuation progress of the ISDEA. h) Luminance of original and healed ISDEA under different AC electric fields.

As presented in **Figure** **4**a,b, the coating of PHT‐IL on both sides of PHT‐ZnS:Cu constructs an ISDEA with a premature breakdown warning function, with color coordinates of (0.1721, 0.3253). The luminescence occurs when an excited electron in the conduction band is subsequently trapped by shallow donor levels under the effect of an electric field and recombines with Cu impurity.^[^ ^62^ ^]^ From the optical microscopy image of Figure S8 (Supporting Information), the thickness of the PHT‐IL is estimated to be ≈170 µm, while that of PHT‐ZnS:Cu is ≈1 mm. Of note, the interfacial compatibility between the dielectric elastomer and compliant electrode layers is critical for ISDEA. Traditionally, stringent vacuum lamination conditions^[^ ^1^, ^17^, ^63^ ^]^ are required to exclude air between the dielectric elastomer and compliant electrode layers to prevent premature electrical breakdown of DEAs. In this work, PHT‐IL and PHT‐ZnS:Cu layers were easily assembled through the stacking process at 60 °C for 3 h. The maximum bonding stress between PHT‐IL and PHT‐ZnS:Cu reaches 0.08 MPa (Figure 4c), which is close to the maximum tensile stress of PHT‐IL, and the final fracture occurred in PHT‐IL instead of the interface (Video S2). The strong bonding can be attributed to the interdiffusion of PDMS‐HDI‐TFB chains between PHT‐IL and PHT‐ZnS:Cu, as shown in Figure S9 (Supporting Information).^[^ ^64^, ^65^ ^]^

The actuation performances of ISDEA with round compliant electrodes under DC are shown in Figure 4d, and the actuated ISDEA with other different shapes of compliant electrodes are shown in Video S3. A maximum actuated strain of 17.0% is obtained by ISDEA under 15 kV mm^−1^. After healing from mechanical damage, the healed actuated strain reaches 15.6% at 14.5 kV mm^−1^, with a healing efficiency of 91.9%. A comparison of actuation performances between several healable DEAs and this ISDEA is provided in Table S1 (Supporting Information). Compared to previously reported healable DEAs, this ISDEA demonstrates a comprehensive advantage of high actuated strain, relatively low actuated electric field, and excellent healing efficiencies after both mechanical and electrical damage. More importantly, the ISDEA shows the premature breakdown warning function (Video S4), which is not found in other reported healable DEAs. Additionally, the inset photographs clearly show no light emission during actuation until an electrical breakdown occurred, and the momentary light emission concentrated at the defects.^[^ ^66^ ^]^ However, the excited electrons after the electrical breakdown transit to the valence band, releasing energy in the form of luminescence. Therefore, the luminescence phenomenon can be used as a warning to turn off the DC power and protect ISDEA from irreversible electrical breakdown.^[^ ^67^, ^68^ ^]^

The actuation performance of ISDEA under an AC electric field with different frequencies is shown in Figure 4e,f. The actuated strain of ISDEA decreases with the increase in AC frequency, particularly under high electric fields. The reason for this has to do with the elastic hysteresis of PHT‐ZnS:Cu film making the response of ISDEA incapable of catching up with the change in AC frequency. The actuated strain of original and healed ISDEAs at 4 Hz (Figure 4g) reveals a healing efficiency of 98.2% based on the largest actuated strain. Compared to the original ISDEA, almost no decline in luminance intensity is observed in healed ISDEA (Figure 4h).

**Figure 5:**
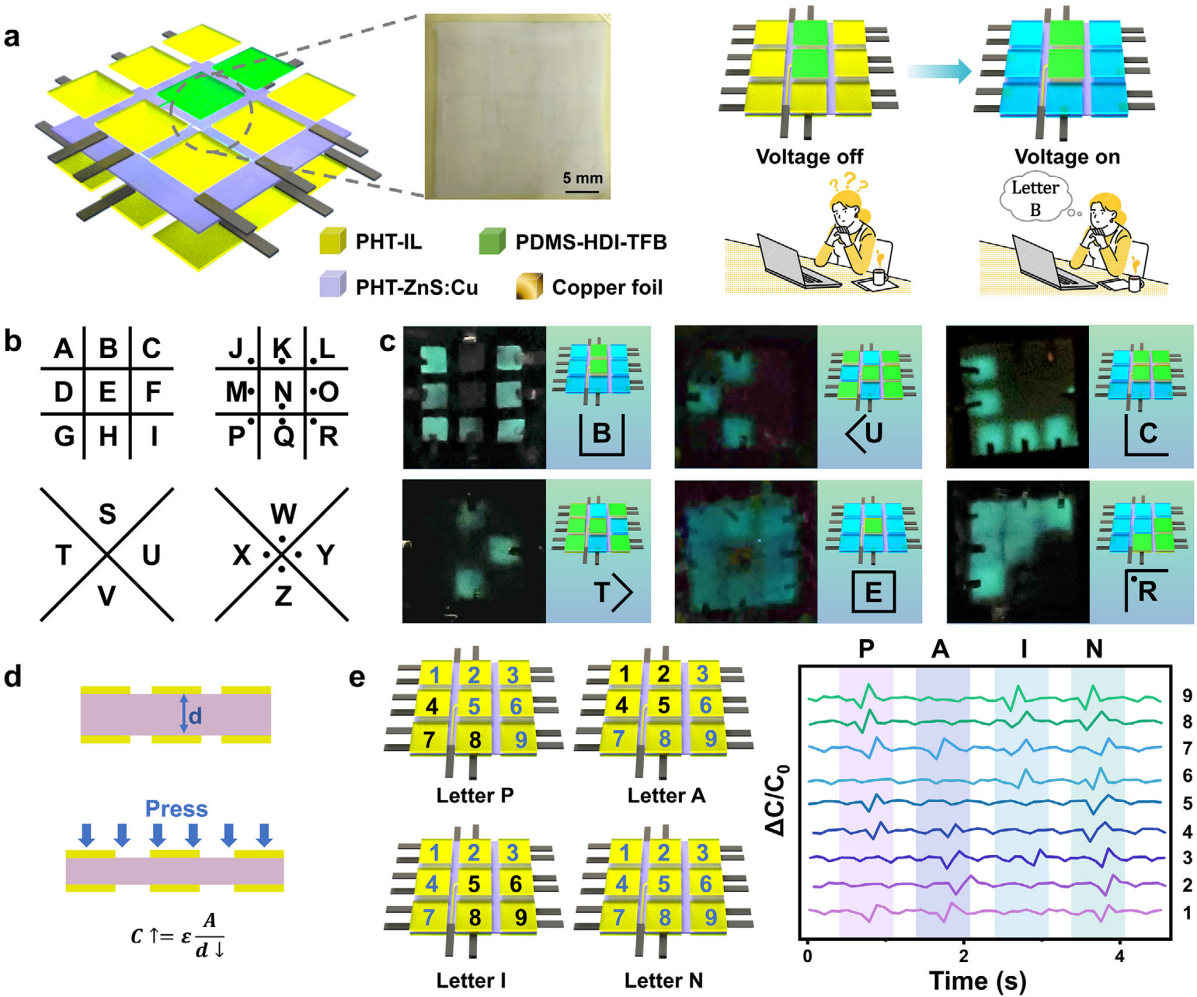
a) Illustration of the construction of the ISDEA soft anti‐counterfeiting label. b) Schematic diagram of the pigpen cipher. c) “BUCTER” was sent by the ISDEA soft anti‐counterfeiting label based on a pigsty password. d) Structure and working principle of the ISDEA soft intelligent keyboard based on a flexible capacitive sensor. e) “PAIN” sent by the ISDEA soft intelligent keyboard with a pigsty password. Note that *C_0_* stands for the initial capacitance, while *ΔC* is the difference between the actual capacitance and the initial capacitance.

As displayed in **Figure** **5**a, a soft anti‐counterfeiting label was constructed by encoding the arrangement of PHT‐IL and PDMS‐HDI‐TFB on several PHT‐ZnS:Cu into a 3 × 3 array. The anti‐counterfeiting functionality is achieved by selectively covering the top and bottom surfaces of the PHT‐ZnS:Cu layer with either PHT‐IL or PDMS‐HDI‐TFB to form distinct patterns that encode information. These two materials are visually indistinguishable without external stimulation. However, upon applying an external voltage, only the regions covered with conductive PHT‐IL can form a complete EL device with PHT‐ZnS:Cu, enabling light emission. In contrast, the regions covered with non‐conductive PDMS‐HDI‐TFB fail to form an effective electrical circuit, thus remaining dark. The soft anti‐counterfeiting label can be applied as a security mark for protecting high‐value merchandise and preventing infringement. According to the pigpen cipher correspondence (Figure 5b), the ISDEA‐based soft anti‐counterfeiting labels display the letters “B,” “U,” “C,” “T,” “E,” and “R” (Figure 5c, Video S5).

Moreover, the ISDEA can be directly used as a flexible capacitive sensor, in which the classic plate capacitor calculation method (*C* = *ε*_0_ *ε*_r_ *S*/*d*) is employed for calculations (Figure 5d). Here, *C*, *ε*_0_, *ε*_r_, *S*, and d stand for the capacitance, vacuum permittivity, relative permittivity, plate area, and dielectric layer thickness of the dielectric elastomer, respectively. Under mechanical stimuli such as pressing or bending, the gauge factor of the flexible capacitive sensor (defined as ΔC/C_0_) increases due to the reduced distance between the two electrodes, which leads to an increase in capacitance. Thus, the ISDEA‐based capacitive sensor array can be utilized as a soft intelligent keyboard. During an emergency, the ISDEA‐based flexible capacitive sensor sends out patients’ requirements in real time or human emotion secretly, such as “PAIN” (Figure 5e) and “HELP” (Figure S10, Supporting Information) based on the pigpen cipher. In this process, the “PAIN” signal is sent out by pressing the sensor, while the “HELP” signal is sent out by stretching the sensor, resulting in different peak shapes. The as‐constructed ISDEA‐based soft anti‐counterfeiting labels and a soft intelligent keyboard have broad and promising application prospects in ensuring reliable archival records, detecting counterfeit goods, and helping information transmission secretly.

## 3 Conclusion

An ISDEA with a premature breakdown warning function was successfully prepared by synthesising a healable poly(dimethylsiloxane) based on reversible imine bonds and hydrogen bonds, and subsequent incorporation ofZnS:Cu particles. When the applied electric field is close to the breakdown strength of DEAs, the ZnS:Cu particles in dielectric elastomers emit light, providing a visible warning to prevent premature electrical breakdown. The introduction of LiTFSI into the PDMS‐HDI‐TFB forms a healable transparent compliant electrode suitable for clear observation of the light emission of ZnS:Cu particles without interference. The as‐formed ISDEA shows an actuated strain of 17.0% at 15 kV mm^−1^, with 91.9% healing efficiency. By encoding the arrangement of ISDEAs, a soft anti‐counterfeiting label was constructed based on EL property to prevent infringement. Moreover, an ISDEA‐based soft intelligent keyboard relying on capacitive changes was prepared to facilitate information transmission and emotional expression. Such soft anti‐counterfeiting labels and soft intelligent keyboards have potential applications in personal privacy and human‐machine interaction.

## 4 Experimental Section

### Materials

TFB was obtained from Adamas (China), and HDI was produced by Macklin (China). N‐PDMS (Mn = 10000) was received from Heowns (China), and LiTFSI was provided by Sigma–Aldrich (America). Carbon grease, VHB tape, and ZnS:Cu particles were all purchased from a local market.

### Preparation of PDMS‐HDI‐TFB

The synthesis process consisted of first dispersing 5 g of N‐PDMS and different amounts of HDI (80, 72, 64, and 56 µL) in 10 mL of tetrahydrofuran (THF). After mechanical stirring for 12 h under room temperature, different amounts of TFB (0, 8.5, 16.9, and 25.4 mg) were added to the above mixture and continuously stirred for 4 h. The obtained mixture was poured into a polytetrafluoroethylene (PTFE) mould and dried at room temperature for 12 h to obtain the healable PDMS‐HDI_x_‐TFB_1‐x_ (where ‘x’ and ‘1‐x’ represent the mole fraction of HDI and TFB, respectively).

### Preparation of PHT‐IL

The formation process consisted of first adding 2 g of LiTFSI and 5 g of N‐PDMS into 3 mL of THF. Next, 64 µL of HDI was added to the mixture and stirred for 10 h, followed by the addition of 16.9 mg of TFB and stirring for 2 h. Afterward, the mixture was poured into a PTFE mould and volatilized in a vacuum oven at room temperature to yield the PHT‐IL film.

### Preparation of PHT‐ZnS:Cu

The preparation process consisted of first adding 5 g of N‐PDMS and 64 µL of HDI into the 3 mL THF under stirring for 12 h. Afterward, 16.9 mg of TFB and 2 g of ZnS:Cu were introduced into the above mixture and stirred for 4 h. After completion, the reaction products were poured into a PTFE mould and volatilized in a vacuum oven to obtain PHT‐ZnS:Cu films.

### Characterization

A Bruker‐Invenio‐S FT‐IR spectrometer (Germany) was used to analyse the chemical structures of the materials. Further structural analysis was obtained by a ^1^H NMR spectrometer (400 MHz, Bruker Ultrashield 400 Plus, Germany). The mechanical damage of the samples was produced by using a sharp blade, and the healing process was observed using a Nikon E100 optical microscope (Japan). The mechanical properties of samples were measured on a UTM‐2460 universal testing machine (China) at a crosshead speed of 50 mm min^−1^. The UV–vis spectra were collected by Shimadzu UV‐2550 equipment (Japan), and the thickness of PDMS‐HDI_0.8_‐TFB_0.2_ and VHB are both 1 mm. The dielectric properties were collected on the Novocontrol Concept 50 instrument (Germany). The cross‐section fracture morphologies and surface elements of the PHT‐IL and PHT‐ZnS:Cu were identified by a ZEISS SUPRA55 SEM (Germany) equipped with an EDS module. The crosslinking density of the samples was evaluated using the equilibrium swelling method.^[^ ^69^ ^]^ The ionic conductivity of PHT‐IL was measured by an electrochemical workstation (CHI660E, USA) in the frequency range of 1 Hz–1 MHz, and an AC amplitude of 10 mV. The ionic conductivity (*σ*) was calculated as follows:

(1) $$\sigma =L/{\mathrm{WtZ}}^{\prime }$$

where *L* represents the distance between the electrodes, *W* is the length of the electrode, *t* refers to the thickness of the PHT‐IL, and *Z’* is the real component of impedance measured at the minimum phase angle frequency, respectively.^[^ ^70^ ^]^ The color coordinates of PHT‐ZnS:Cu was measured by a JASCO FP‐8550 spectrofluorometer (Japan). The DC voltage was obtained by a Boer 71 230 high‐voltage power supply (China), and the AC power supply was provided by a RIGOL DG1022U DG1000 arbitrary waveform function generator (China) and an Aigtek ATA‐7050 high‐voltage amplifier (China). The luminance of ISDEA was measured by an AccuMAX XRP‐3000 light meter (USA). The changes in capacitance of the ISDEA‐based flexible capacitive sensor were recorded by a Keithley DAQ 6510 (USA). After cutting or electrical breakdown, healing tests of ISDEA were carried out after healing for 3 h at 60°C. The healing efficiency was determined as follows:

(2) $$H=S_{O}/S_{H}\times 100\%$$

where *H* represents the healing efficiency, *S*_O_ is the functional value of the original ISDEA, and *S*_H_ refers to the functional value of healed ISDEA, respectively.

### Volunteer Consent Statement

The experiments involving human subjects were performed with the full consent of the participants and were approved by the ethics committee of the Beijing University of Chemical Technology.

## Conflict of Interest

The authors declare no conflict of interest.

## Supporting information

Supporting Information

Supplemental Video 1

Supplemental Video 2

Supplemental Video 3

Supplemental Video 4

Supplemental Video 5

## Data Availability

The data that support the findings of this study are available from the corresponding author upon reasonable request.
