# Supplementary material for: Healable Dielectric Elastomer Actuator With Premature Breakdown Warning Function for Information Transmission Encryption and Human‐Machine Interaction
Source: Adv Sci (Weinh). 2025 Jun 25;12(35):e05829. doi: 10.1002/advs.202505829 (PMC12463011; doi:10.1002/advs.202505829)
Supplement: Supplementary file 1 — Supporting Information [file ADVS-12-e05829-s006.docx]

**Supporting Information**

**Healable dielectric elastomer actuator with premature breakdown warning function** **for information transmission encryption and human-machine interaction**

Yanze Liu^1^, Shilin Luo^1^, Jiawei Zhao^1^, Lei Xu^1^, Zhong-Zhen Yu^1,2*^, Dan Yang^1,2*^

^1^State Key Laboratory of Organic-Inorganic Composites, College of Materials Science and Engineering, Beijing University of Chemical Technology, Beijing 100029, China

^2^Center for Nanomaterials and Nanocomposites, College of Materials Science and Engineering, Beijing University of Chemical Technology, Beijing 100029, China

*E-mails: yuzz@mail.buct.edu.cn (Z.-Z. Yu); danyang@buct.edu.cn (D. Yang)


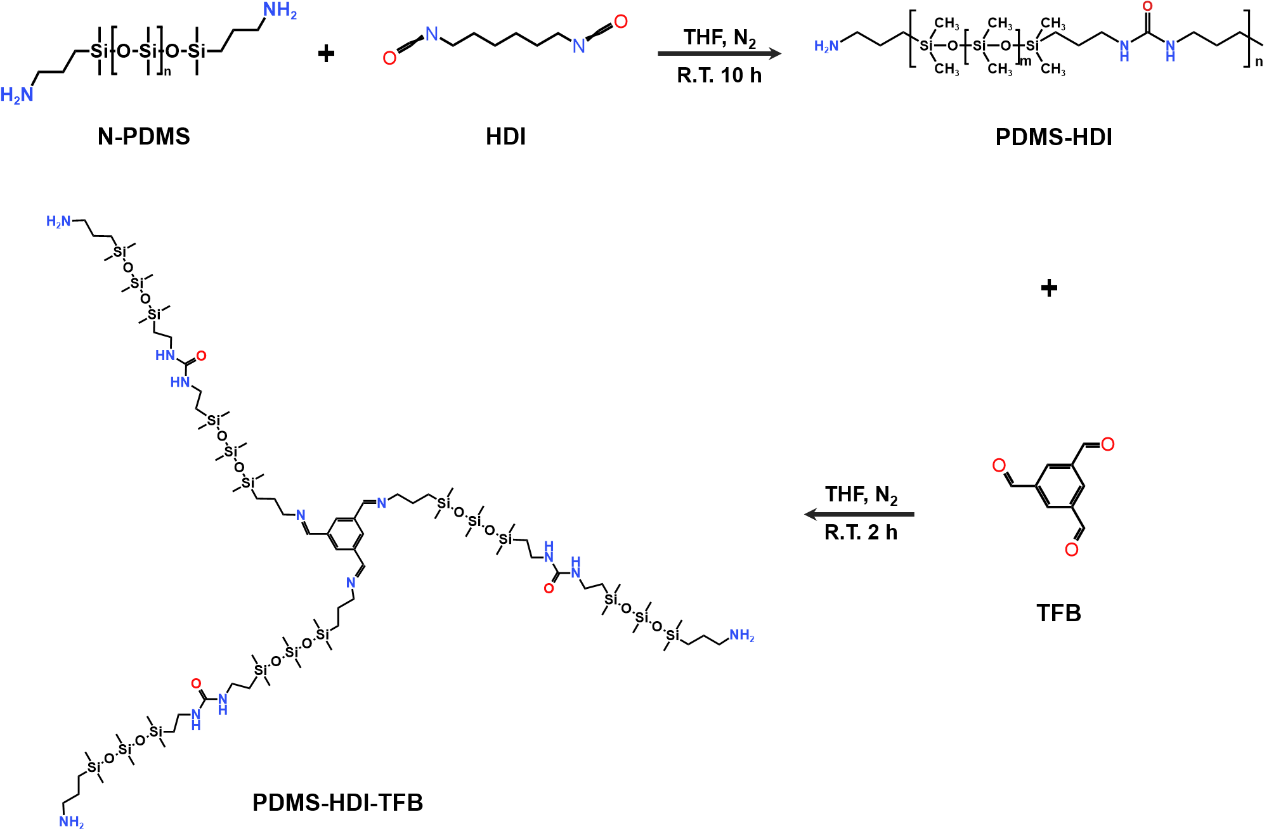


**Figure S1.** Synthetic route of the PDMS-HDI-TFB.

**Figure S2.** Young’s modulus of PDMS-HDI_x_-TFB_1-x_.


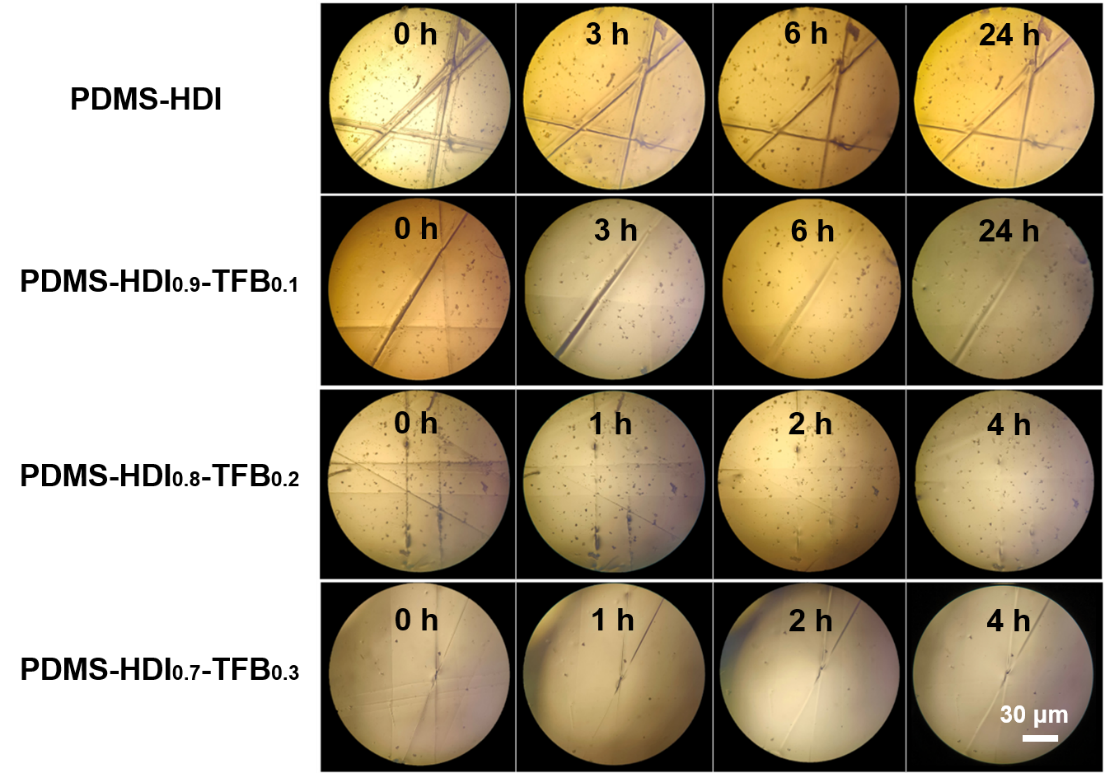


**Figure S3.** Photographs of healing progress of PDMS-HDI, PDMS-HDI_0.9_-TFB_0.1_, PDMS-HDI_0.8_-TFB_0.2_, and PDMS-HDI_0.7_-TFB_0.3_ under optical microscopy.


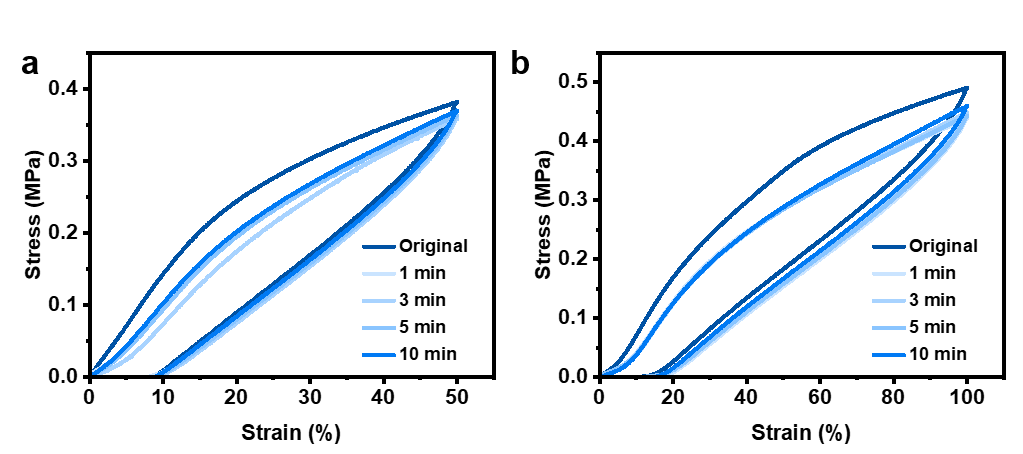


**Figure S4.** Cyclic loading–unloading tensile curves obtained by repeating the (a) 50% and (b) 100% stretching ratio with different rest intervals between each cycle.


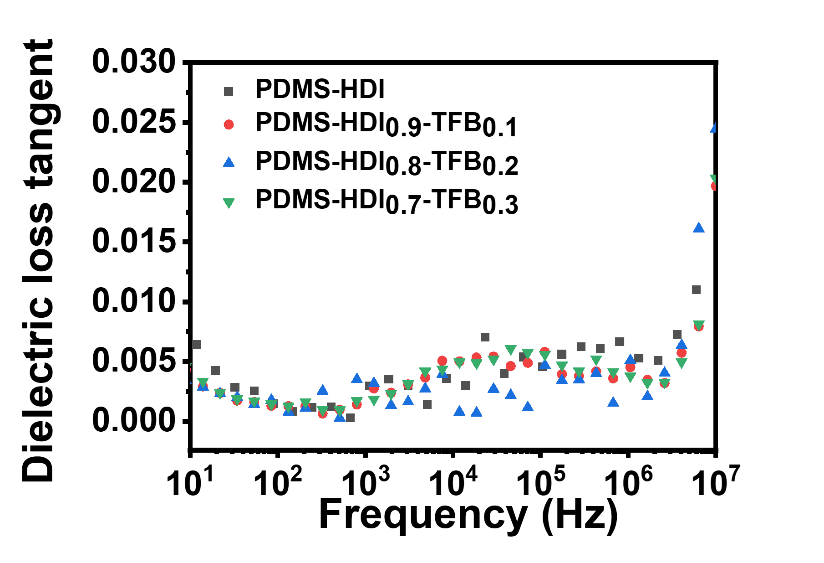


**Figure S5.** Dielectric loss tangent of PDMS-HDI_x_-TFB_1-x_ versus frequency.


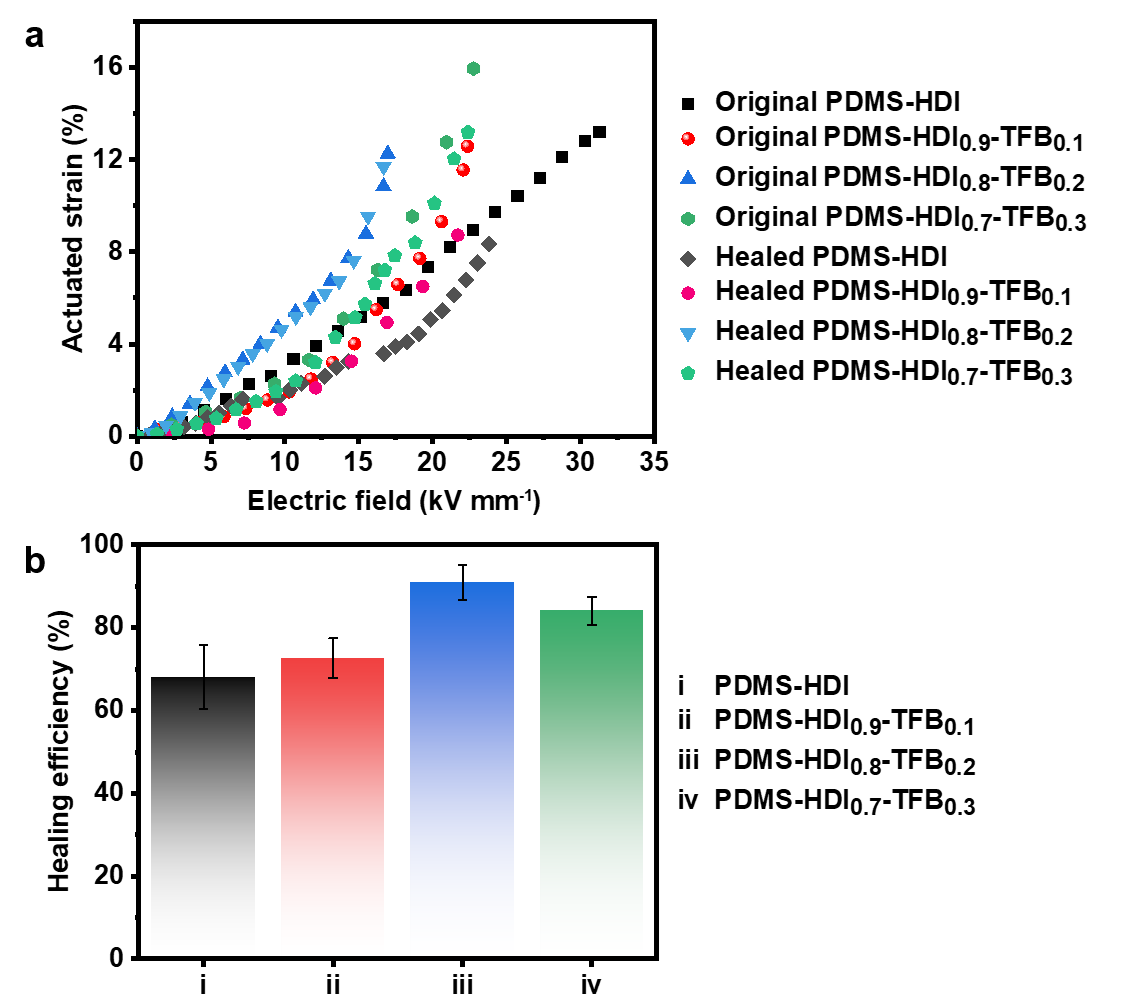


**Figure S6.** (a)Actuated strains of the original and healed PDMS-HDI_x_-TFB_1-x_ after mechanical damage. (b) Healing efficiency of the PDMS-HDI_x_-TFB_1-x_ based on the largest actuated strain after mechanical damage.

**
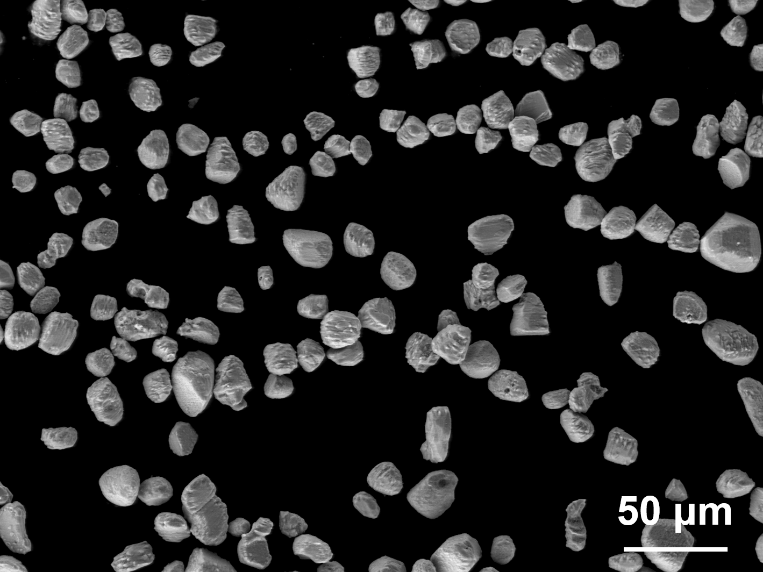
**

**Figure S7.** SEM image of ZnS:Cu particles.


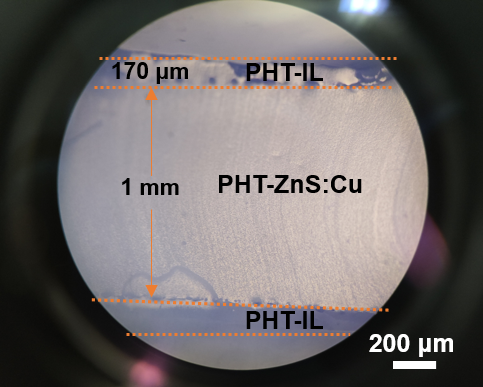


**Figure S8.** A photograph of ISDEA under optical microscopy.


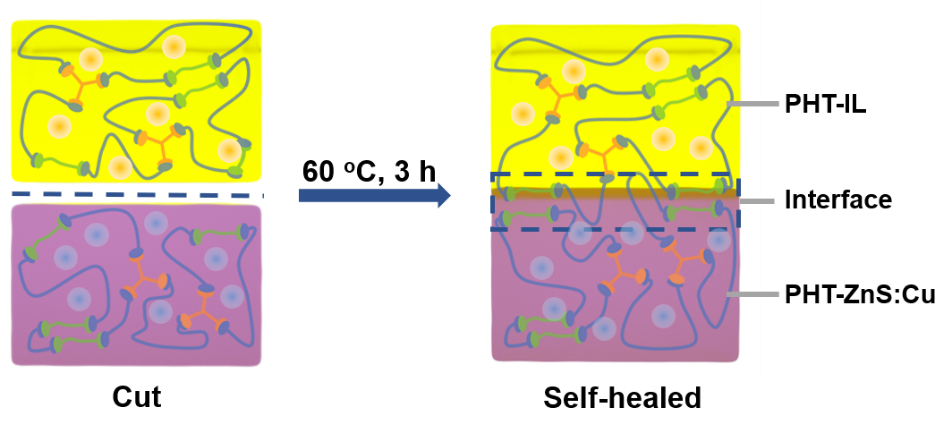


**Figure S9.** Illustration of the interdiffusion of PDMS-HDI-TFB chains between PHT-IL and PHT-ZnS:Cu.


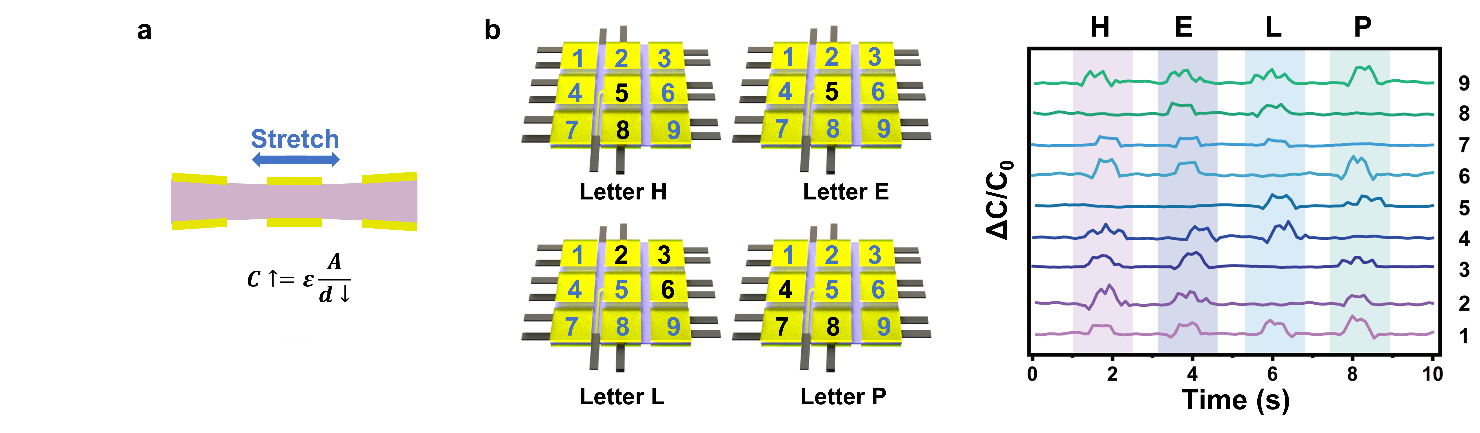


**Figure S10.** (a) The working principle of the ISDEA soft intelligent keyboard based on a flexible capacitive sensor by stretching. (b) “HELP” sent by the ISDEA soft intelligent keyboard with a pigsty password.

**Table S1.** Comparison of actuation performances and healing efficiency between DEAs in this work with other healable DEAs.

**Videos：**

**Video S1** Stretching and twisting of PDMS-HDI-TFB.

**Video S2** Adhesion test between PHT-IL and PHT-ZnS:Cu.

**Video S3** Actuated ISDEA with different shapes of compliant electrodes.

**Video S4** Actuated performance and premature breakdown warning function of ISDEA.

**Video S5** Display of “B”, “U”, “C”, “T”, “E”, and “R” letters by actuating ISDEA soft anti-counterfeiting label.

**References:**

[1] Zhang Y., Ellingford C., Zhang R., Roscow J., Hopkins M., Keogh P., et al., Electrical and Mechanical Self‐Healing in High‐Performance Dielectric Elastomer Actuator Materials, 2019, Adv. Funct. Mater., 29, 1808431, https://doi.org/10.1002/adfm.201808431.

[2] Duan L., Lai J. C., Li C. H., Zuo J. L., A Dielectric Elastomer Actuator That Can Self-Heal Integrally, 2020, ACS Appl. Mater. Interfaces, 12, 44137, https://doi.org/10.1021/acsami.0c11697.

[3] Peng Z., Shi Y., Chen N., Li Y., Pei Q., Stable and High‐Strain Dielectric Elastomer Actuators Based on a Carbon Nanotube‐Polymer Bilayer Electrode, 2020, Adv. Funct. Mater., 31, 2008321, https://doi.org/10.1002/adfm.202008321.

[4] Liu L., Zhang W., Ning N., Zhang L., A Self-Healing Dielectric Supramolecular Elastomer Modified by TiO_2_/urea Particles, 2019, Chem. Eng. J., 375, 121993, https://doi.org/10.1016/j.cej.2019.121993.

[5] Sun H., Liu X., Liu S., Yu B., Ning N., Tian M., Zhang L., Silicone Dielectric Elastomer with Improved Actuated Strain at Low Electric Field and High Self-Healing Efficiency by Constructing Supramolecular Network, 2020, Chem. Eng. J., 384, 123242, https://doi.org/10.1016/j.cej.2019.123242.

[6] Tan M. W. M., Thangavel G., Lee P. S., Rugged Soft Robots using Tough, Stretchable, and Self‐Healable Adhesive Elastomers, 2021, Adv. Funct. Mater., 31, 2103097, https://doi.org/10.1002/adfm.202103097.

[7] Tan M. W. M., Bark H., Thangavel G., Gong X., Lee P. S., Photothermal Modulated Dielectric Elastomer Actuator for Resilient Soft Robots, 2022, Nat. Commun., 13, 6769, https://doi.org/10.1038/s41467-022-34301-w.

[8] Liu L., Yan S., Zhang L., A Self‐Healing Dielectric Supramolecular Elastomer Functionalized with Aniline Tetramer, 2018, Rapid Commun., 39, 1800349, https://doi.org/10.1002/marc.201800349.
